# Unmasking of Myasthenia Gravis After Introduction of Oral Risperidone in a Schizophrenic Saudi Male: A Case Report

**DOI:** 10.7759/cureus.20541

**Published:** 2021-12-20

**Authors:** Lamees Abualkhair, Ahmad Almaghrabi, Nada Al Edrees, Ahmed Hassab Errasoul

**Affiliations:** 1 Department of Psychiatry, College of Medicine, King Saud University, Riyadh, SAU; 2 College of Medicine, Alfaisal University, Riyadh, SAU; 3 Department of Psychiatry, Eradah Hospital and Mental Health, Jazan, SAU

**Keywords:** adverse effects, extrapyramidal side effects, antipsychotics, schizophrenia, risperidone, myasthenic exacerbation, myasthenia gravis

## Abstract

Myasthenia gravis is an autoimmune disease that affects the neuromuscular junction. It causes generalized muscle weakness that may include the respiratory muscles, potentially leading to a medical emergency known as a myasthenic crisis. This is a case report of a rare incident of a myasthenia gravis exacerbation after administration of the oral antipsychotic risperidone to a schizophrenic patient. Few similar cases have been reported. Although rare, such incidents are dangerous as physicians could easily confuse myasthenia gravis symptoms with extrapyramidal side effects of antipsychotics. This concern should be addressed not just with risperidone but rather with any other antipsychotics that exhibit anticholinergic side effects.

## Introduction

Myasthenia gravis is a rare autoimmune disorder with incidence and prevalence of 0.3-2.8 and 5.35-35 per 100,000, respectively [1]. It is caused by autoantibodies that target the postsynaptic acetylcholine receptors (AChR) in the neuromuscular junction, leading to muscle weakness and fatigability, and could lead to a life-threatening myasthenic crisis with respiratory depression that requires urgent assisted ventilation [2]. The classic symptom is a fluctuating muscular weakness that worsens progressively throughout the day [2]. Infections, vaccines, surgeries, emotional stress, and drugs are examples of exacerbating factors [2].

Schizophrenia is a devastating psychiatric disorder with a prevalence rate of 1% [3]. It is characterized by variable signs and symptoms including positive and negative symptoms which affect cognition, emotion, perceptions, and behavior [3]. Antipsychotic medications are the mainstay of the management of schizophrenia and many agents in this drug class are with varying degrees of anticholinergic side effects [4]. In this rare occasion of comorbid myasthenia gravis in a patient with psychotic disorder needing antipsychotics for management, serious exacerbations could be life-threatening causing severe respiratory depression due to the depletion of the neurotransmitter acetylcholine stores in critical sites across the body’s neuromuscular junctions such as the intercostal and diaphragmatic muscles [2]. Here, we report a case of a 20-year-old Saudi male diagnosed with schizophrenia with no previous history of myasthenia gravis or neurological disorder nor a family history of neurological disorder, who had a new onset of myasthenia gravis after an introduction of oral risperidone.

## Case presentation

A 20-year-old Saudi male presented to the emergency department against his will due to a one-and-a-half-year history of persecutory delusions towards his family, disorganized behavior, disorganized speech, and irritability. His symptoms were associated with poor oral intake and as a result deterioration in the level of functioning in academic, social, and self-care. He was diagnosed with schizophrenia and admitted to the psychiatric ward to stabilize his condition. He had no previous history of psychiatric or neurological disorder as well as no family history of neurological or psychiatric disorder. He was started on risperidone oral 2 mg daily for around three weeks. During his admission, he started to complain of difficulty in opening his eyelids and was thought to be an extrapyramidal side effect due to risperidone and procyclidine 5 mg; an anticholinergic was prescribed when necessary (pro re nata {PRN}) due to assumption that the difficulty to open eyelid was an extrapyramidal side effect (EPS). He then started to show muscle rigidity, sialorrhea (saliva drooling), and dysphagia. He was consuming procyclidine 5 mg twice a day (bis in die {BID}) to alleviate the symptoms. His medication was crossly titrated to olanzapine 5 mg after 12 days of admission, with no resolution of dysphagia. The ears, nose, and throat (ENT) and swallowing teams were consulted for assessment. Examination showed that he had soft palate weakness with uvular deviation and their recommendations were to keep the patient on intravenous (IV) fluid for hydration, to be kept nothing by mouth (NPO), and to insert a nasogastric tube (NGT) for feeding. Computerized tomography (CT) of the neck and soft tissues was done, and results were negative for laryngeal pathology or suspicious cervical lymphadenopathy. CT brain revealed right frontal subcortical nonspecific focal hypodensity. His condition deteriorated and developed hoarseness of voice. Neurology team was consulted and recommended for magnetic resonance imaging (MRI) brain and cervical spine with contrast, thyroid panel, vasculitis panel to send out acetylcholine receptor antibodies, spirometry every four hours, pulmonary function test, CT chest with contrast, repetitive nerve stimulation, and trial of pyridostigmine 60 mg every six hours. The initial impression was acute bulbar syndrome. Transfer of care of the patient was addressed and his care was transferred under the neurology team. An NGT was inserted and feeding was reinitiated. MRI brain showed round enhanced nonspecific lesion in the corpus callosum.

The autoimmune panel showed high erythrocyte sedimentation rate (ESR) and c-reactive protein (CRP). CT chest showed signs of aspiration pneumonia. The lumbar puncture (LP) was negative. The pulmonology team was consulted and their assessment was chronic chest CT secondary to chronic aspiration and no need to be started on antibiotics. He has been placed on pyridostigmine 60 mg every six hours. Neurological examination revealed left eye ptosis, bilateral lower motor neuron facial weakness, absent gag reflex, uveal deviated to the right side, decreased movement in the right palate, and nasal speech. Repetitive stimulation performed on the ulnar and facial nerves revealed 33% and 35% decrement in compound muscle action potential amplitudes, respectively. No facilitation was present following 10 seconds of isometric contraction. Partial repair of decrement was present at two minutes post one minute of isometric exercise in the abductor digiti minimi, interpreted as there is electrodiagnostic evidence of a postsynaptic neuromuscular junction disorder that would be compatible with the clinical suspicion for myasthenia gravis. Intravenous immune globulin (IVIG) was initiated for five days as well as Cellcept 500 mg BID and prednisolone 40 mg oral daily. The physiotherapy team was involved in his care. After the third day of IVIG, ptosis and weakness started to improve. Repeated CT for suspected thymoma was negative. IVIG course completed, resolved ptosis and weakness. However, he still had dysphagia, nasal speech, and absent gag reflex. Plasmapheresis was started, after which dysphagia and nasal speech improved. He continued on olanzapine 7.5 mg oral daily with good response, and due to him being drug-naive, he needed a small dose to control his behavior and had no signs of deterioration or exacerbation of myasthenia gravis. He was discharged safely on pyridostigmine 60 mg oral four times a day, prednisolone 35 mg daily. Acetylcholine receptor (AChR) antibody titer was 0.03 nmol/L and muscle-specific tyrosine kinase (MuSK) titer was <0.2 U/mL. Neurology team's final impression was myasthenia gravis, and the patient is receiving collaborative care between psychiatry and neurology.

## Discussion

Myasthenia gravis and schizophrenia are both rare diseases with a rarest comorbidity. Management of this comorbidity can be challenging due to the scarcity of literature, overlapping symptomatology, and conflicting pharmacodynamics. The lifetime prevalence of one or more autoimmune diseases is increased by 50% in schizophrenia [5], hence schizophrenia poses a possible risk factor of developing myasthenia gravis [6]. Till present day, the comorbidity of both disorders has been reported in around 10 cases that had varying degrees of myasthenic exacerbations [7].

Regardless of the unknown underlying mechanism of this comorbidity and its rare incidence, the potentially severe exacerbations of administered psychotropics and the significant overlapping of symptoms make myasthenia gravis a challenging yet critical diagnosis for psychiatrists [8,9]. Several routinely used psychotropics (antipsychotics, mood stabilizers, and antidepressants) have been reported to exacerbate myasthenia gravis, mainly due to its anticholinergic properties [9]. Some of their extrapyramidal symptoms that overlap with myasthenia gravis symptoms are difficulty in breathing, swallowing and speech, and slowing of motor function in extremities [10-12]. Although the anticholinergic effect of antipsychotic medications is mainly at muscarinic receptors, some have limited but significant activity at nicotinic receptors as well [4]. Agents in the antipsychotic class that reported such interaction include chlorpromazine, clozapine, olanzapine, sulpiride, risperidone, pimozide, thioridazine, haloperidol, quetiapine, and zuclopenthixol acetate [13,14].

Risperidone is a second-generation antipsychotic (SGA) that functions by antagonizing D2 and 5HT2A receptors, and it is administered in an oral form (tablets, solution, or dissolvable M-TABs) or as a long-acting injection. The Food and Drug Administration (FDA) approved indications for oral risperidone (tablets, oral solution, and M-TABs) include the treatment of schizophrenia, bipolar I acute manic or mixed episodes, and autism-associated irritability [15].

Our patient was on 2 mg oral risperidone. During his admission, he developed ptosis which was mistaken for extrapyramidal symptoms and so was put on the anticholinergic procyclidine which further worsened his symptoms. He developed muscle rigidity, sialorrhea, dysphagia, and hoarseness of voice. This deterioration needed a five-day course of IVIG and plasmapheresis before showing improvement in his symptoms.

A review of the literature of case reports published in English in PubMed, Scopus, and Google scholar showed two previous case reports of myasthenia exacerbation on risperidone, a 29-year-old schizophrenic female patient from Kuwait taking the injectable form [16] and a six-year-old female with attention deficit hyperactivity disorder (ADHD) from turkey taking the oral form [8]. In terms of severity of the exacerbation after risperidone administration, it ranged from ptosis and weakness to severe respiratory depression requiring ICU admission [8,16].

Further research is required to explore this effect of risperidone concerning its anticholinergic properties specifically in the light of myasthenia gravis comorbidity as it could be life-threatening to patients with schizophrenia or any psychotic disorder. Psychiatrists need to be clinically aware of the overlap between myasthenia and extrapyramidal side effects and to be suspicious of underlying neurological disorders when no improvement of traditional management has been achieved.

## Conclusions

Psychotropic choice in patients with myasthenia gravis can be challenging due to their anticholinergic properties that can exacerbate myasthenia gravis symptoms with potential deterioration to a myasthenic crisis. And due to the overlapping between extrapyramidal side effects and myasthenia gravis symptoms, diagnosis of myasthenia gravis and appropriate intervention can be delayed. Further research is needed to explore potentially critical exacerbations on myasthenia gravis and its relation with psychotropics to determine their safety and efficacy.
